# Nanoscale Flexibility Parameters of Alzheimer Amyloid Fibrils Determined by Electron Cryo-Microscopy**

**DOI:** 10.1002/anie.200904781

**Published:** 2010-01-12

**Authors:** Carsten Sachse, Nikolaus Grigorieff, Marcus Fändrich

**Affiliations:** Max-Planck Research Unit for Enzymology of Protein Folding and Martin-Luther University Halle-WittenbergWeinbergweg 22, 06120 Halle an der Saale (Germany); MRC Laboratory of Molecular BiologyCambridge, (UK); Rosenstiel Basic Medical Sciences Research Center and Howard Hughes Medical Institute, Brandeis UniversityWaltham, MA (USA)

**Keywords:** Alzheimer’s disease, amyloids, electron microscopy, nanotechnology, protein folding

Amyloid fibrils are fibrillar polypeptide aggregates consisting of a cross-β structure.^1^, ^2^ The rigidity and stability of these fibrils contributes to their natural pathogenicity or functionality and has suggested potential applications in bionanotechnology.^3^–^6^ Yet, amyloid fibrils can occur in different morphologies with unique mechanical and flexible characteristics.^7^–^9^ Herein, we use electron cryo-microscopy (cryo-EM) to characterize these nanoscale structural properties. Cryo-EM images effectively represent snapshots of thermally fluctuating fibrils in solution; it is not necessary to micromanipulate or immobilize the fibrils on a solid surface. The amyloid fibrils analyzed here consist of Alzheimer’s Aβ(1–40) peptide. They are homogenous in width (*w*≍20 nm), although different fibrils can vary significantly in their crossover distances *d* (Figure 1).

**Figure 1:**
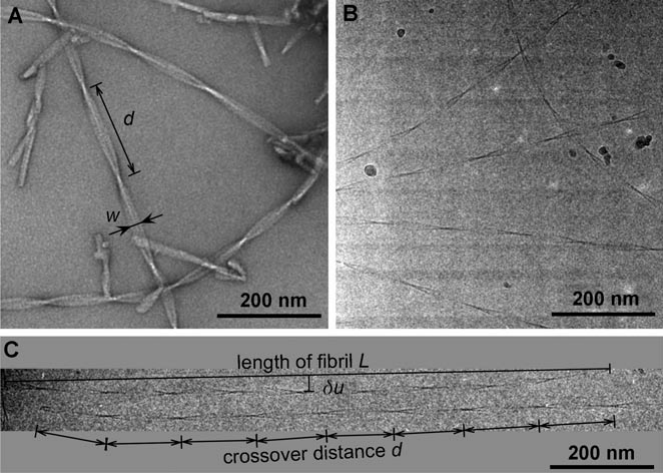
Global structural characteristics. Negatively stained micrographs (A) and cryo-micrographs (B, C) illustrate definitions of fibril length *L*, width *w*, crossover distance *d*, and normal distance *δu*.

In addition to these interfibrillar differences, there are variations of *d* within each single fibril. However, the intrafibrillar standard deviations of *d* range mostly from 5 to 7 nm, while average *d* values of different fibrils vary from 100 to 160 nm (Figure 2 A). Hence, the encountered variations cannot be explained by purely thermally determined and stochastic fluctuations. Instead, they represent subtle, yet systematic, structural differences between the fibrils in the sample.

**Figure 2:**
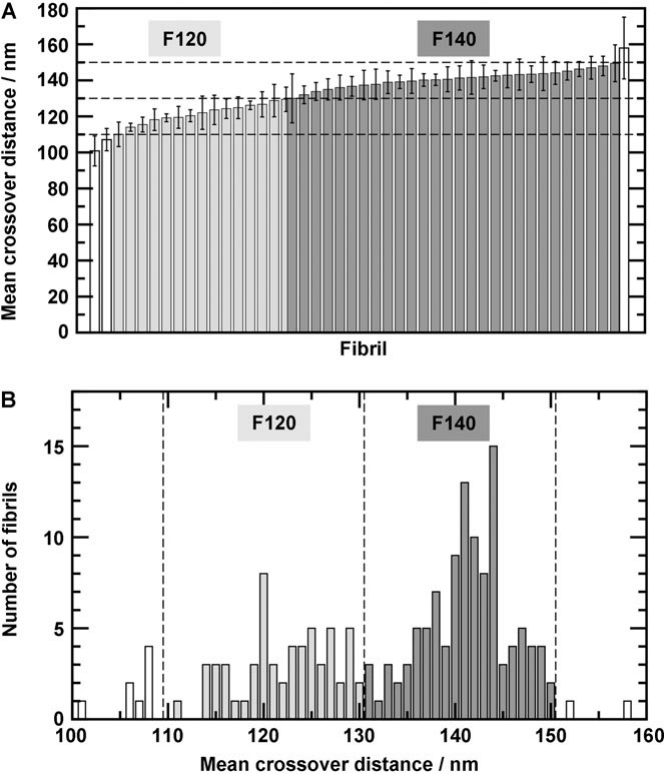
A) Mean crossover distances of representative fibrils. B) Distribution of mean crossover distances of the entire fibril population. F120: light gray; F140: dark gray.

To further analyze these structural differences, two subpopulations were defined, termed here F120 and F140 fibrils. F140 fibrils show mean *d* values of (140±10) nm (Figure 2 B), and their 3D structure was reconstructed previously at approximately 8 Å resolution.^10^, ^11^ F120 fibrils possess an average *d* value of (120±10) nm (Figure 2 B). The structure of F120 fibrils is determined here at approximately 10 Å resolution (Figure 3 A, B, Figure 2 in the Supporting Information). Whereas the distinction between F120 and F140 fibrils remains arbitrary, the two subpopulations consist of a sufficiently large data set for a medium-resolution 3D reconstruction and for measurement of the nanoscale elastic properties. Reconstructed F120 and F140 fibrils present effectively the same cross-section (Figure 3). Hence, the conformational differences of the peptides forming F120 and F140 fibrils are too small to be revealed at the current levels of structural resolution. These data imply that the systematic variations in the crossover distances of different fibrils (Figure 2 A) occur within fibrils that all belong to the same basic morphology. In other words, different fibrils of the same morphology can occur with different torsional properties.

**Figure 3:**
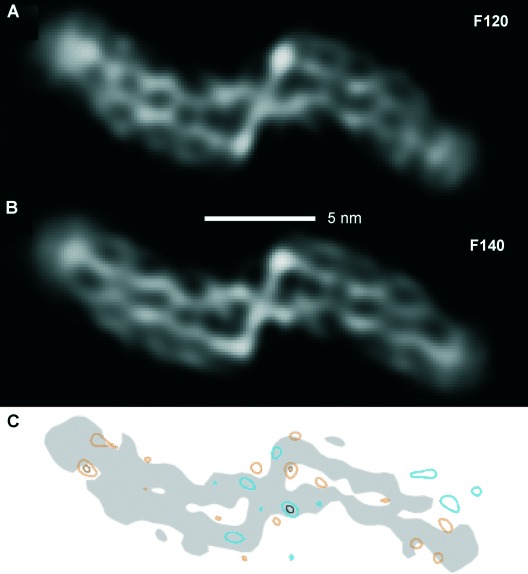
Cross-section of F120 (A) and F140 fibrils (B). C) Difference map F140−F120. Negative peaks: orange=2*σ*, red=3*σ*. Positive peaks: light blue=2*σ*, blue=3*σ*.

Calculation of the nanoscale elastic properties is based on the measurement of variations of fibril twisting and bending. Assuming that the fibrils are made up of an isotropic homogeneous medium, variations of the fibril twist *d* enable computation of torsional persistence length *l*_c_ and torsional rigidity *c*. Bending variations yield persistence length *l*_p_ and bending rigidity *κ* (see the Supporting Information for details). Our measurements imply that F120 and F140 fibrils possess very similar, if not identical, torsional properties (torsional rigidity *c* and torsional persistence length *l*_c_; Table 2 in the Supporting Information). By contrast, the two fibril populations differ significantly in their bending properties (Table 3 in the Supporting Information). F120 fibrils possess a smaller bending rigidity *κ* (Table 3 in the Supporting Information) and a larger normalized bending fluctuation Δ*u* than F140 fibrils (Figure 3 B in the Supporting Information). However, part of this difference may result from the different spacing of crossovers in these two populations (Figure 3 in the Supporting Information).

The measured *l*_p_ and *κ* values are within the reported range for other amyloid fibrils.^12^–^14^ They also comply with a fundamental relationship between *l*_p_ and the molecular density (mass per length; Figure 4 A).

**Figure 4:**
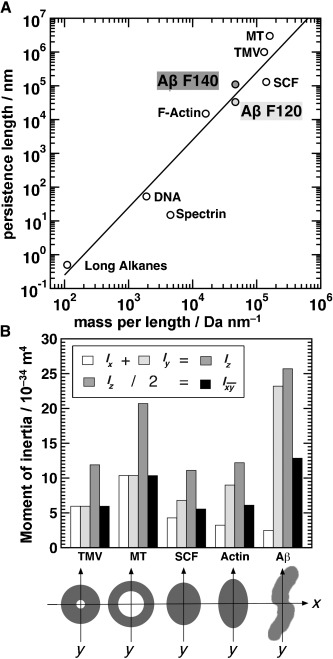
Flexibility parameters of F120 and F140 fibrils in comparison with those of other filamentous protein assemblies. A) Persistence length increases with the increase in mass per unit of length.^19^ B) Comparison of moments of inertia and polar moments of inertia from models of area-normalized cross-sections of different protein filaments. TMV: tobacco mosaic virus, MT: microtubules, SCF: sickle-cell hemoglobin fibers.

For several protein fibrils, the dependence of *c* and *κ* on shape- and material-specific factors has been analyzed.^15^–^17^ The physical formalism used in these analyses was developed for macroscopic objects. Thus, its general applicability to nanoscale protein fibrils remains to be established. According to this formalism, the torsional rigidity *c* depends on the shape-dependent polar moment of inertia *I_z_* and the material-specific shear modulus *G* [Eq. (1)]. The bending rigidity *κ* depends on the material-specific Young’s modulus *Y* and the shape-dependent moment of inertia 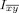 [Eq. (2)].


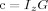
(1)


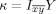
(2)

In contrast to previous approaches that had to use model estimates for the fibril cross-section, cryo-EM enables calculation of the two shape-dependent factors *I_z_* and 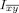 directly from the cross-section of the 3D fibril reconstructions. F120 and F140 fibrils effectively possess the same cross-sectional architecture (Figure 3) and therefore similar shape-specific factors *I_z_* and 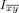 (Tables 2 and 3 in the Supporting Information). The torsional rigidities of F120 and F140 fibrils are very similar and produce the same shear modulus *G* within error margins [Eq. (1), Table 2 in the Supporting Information).

We have compared the calculated material moduli with literature data. Exact numeric values should be considered carefully, however, owing to possible effects of the method of analysis.^14^ The shear moduli *G* of F120 and F140 fibrils (12.7 MPa) are in close proximity to those of other protein assemblies, such as F-actin (9 MPa)^16^ and sickle-cell fibrils (SCF, 1 MPa).^18^ In comparison to macroscopic materials, these values fall in the range between plastics (ca. 100 MPa) and rubber (ca. 0.6 MPa).^19^ The Young’s moduli *Y* of F120 and F140 fibrils (90 and 320 MPa, respectively) are close to the observed values for filamentous proteins, such as SCF (50 MPa),^18^ but are somewhat lower than figures of microtubuli and actin (1 and 3 GPa, respectively).^15^

The material constants of F120 and F140 fibrils differ more profoundly from those reported recently for insulin amyloid fibrils (shear modulus *G*=130 MPa, Young’s modulus *Y*=6 GPa^14^). By contrast, the persistence length (42 μm) and bending rigidity (1.7×10^−25^ N m^2^) of insulin fibrils are remarkably similar to those of Aβ(1–40) fibrils. Since no 3D reconstruction of the analyzed insulin fibrils was reported, their cross-sectional structure cannot be compared easily with the structure of the Aβ(1–40) fibrils used here.

While our data cannot confirm the existence of unusually high nanoscale material constants for the analyzed Aβ(1–40) fibrils, we find that the shape-dependent properties polar moment of inertia *I_z_* and moment of inertia 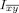 are significantly greater for the analyzed Aβ(1–40) fibrils than for area-normalized cross-sections of other protein filaments (Figure 4 B). Hence, the analyzed Aβ fibrils represent a very material-efficient way to construct proteinaceous filaments of high stability and structural flexibility. These observations are relevant for better estimating the potential applications of amyloid fibrils in the material sciences.

In addition, our data contribute to understanding amyloid pathogenicity in vivo. The stability and flexibility of amyloid fibrils are similar to those of native protein filaments, such as F-actin or microtubules. However, growth and disassembly of the latter represent highly dynamic and regulated processes, and as such they are tightly controlled by specific sets of proteins. Therefore, an unregulated outgrowth of similarly stable amyloid fibrils will be much more difficult to tolerate within a biological environment. This conclusion is consistent with the fact that amyloid pathogenicity arises, at least partially, from the distortion or disruption of naturally elastic and flexible tissues, such as cardiac ventricles or blood vessel walls.^20^ Further work will be required, however, to delineate the cellular pathways by which these reactions result in the death of affected cells.

## Notes

Detailed facts of importance to specialist readers are published as ”Supporting Information”. Such documents are peer-reviewed, but not copy-edited or typeset. They are made available as submitted by the authors.
