# Supplementary material for: Nanoscale Flexibility Parameters of Alzheimer Amyloid Fibrils Determined by Electron Cryo-Microscopy
Source: Angew Chem Int Ed Engl. 2010 Jan 12;49(7):1321–3. doi: 10.1002/anie.200904781 (PMC2913414; doi:10.1002/anie.200904781)
Supplement: Supplementary file 1 [file anie0049-1321-SD1.pdf]

Angewandte Chemie
Eine Zeitschrift der Gesellschaft Deutscher Chemiker

Supporting Information

© Wiley-VCH 2010

69451 Weinheim, Germany

# Nanoscale Flexibility Parameters of Alzheimer Amyloid Fibrils Determined by Electron Cryo-Microscopy**

*Carsten Sachse, Nikolaus Grigorieff, and Marcus Fändrich**

anie_200904781_sm_miscellaneous_information.pdf

## *Sample preparation and electron cryo-microscopy*

Chemically synthetic Aβ(1-40) (Bachem) was dissolved at 5mg/ml concentration in water and snap-frozen in aliquots. To induce fibril formation, one aliquot was thawn and diluted using a 750 mM sodium borate pH 7.8 stock solution to a final concentration of 1 mg/ml peptide at 50 mM borate. The final pH-value obtained upon dilution was 8.7. Fibrils were grown for a minimum of four days and plunge-frozen in the coldroom[1]. An FEI F30 cryo-microscope was operated at 300 kV under low-dose conditions and specimens were imaged on Kodak ISO163 film at a nominal magnification of 59,000.

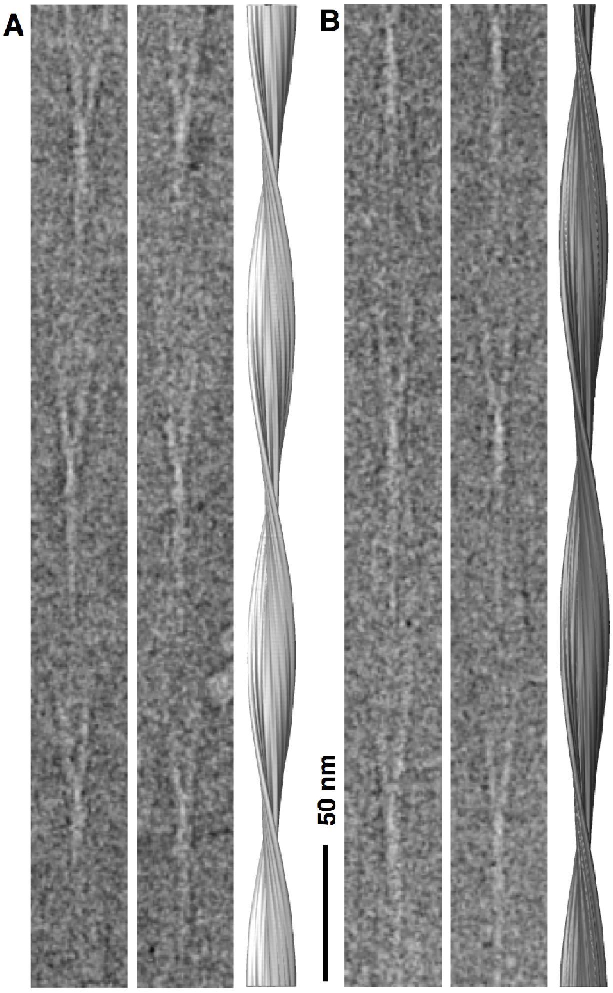


**Supp. Figure 1.** Three-dimensional image reconstruction of Aβ(1-40) amyloid fibrils. (A) and (B) Examples of two straight fibrils from each population included in the image reconstruction. Surface-rendered side view of F120 (light grey) and F140 (dark grey) reconstruction.

## *Image processing and 3D image reconstruction*

We selected 60 micrographs from a total of 250 according to image and fibril quality, and subsequently scanned them on a Zeiss SCAI scanner with a raster size of 7 μm[1]. We assessed fibril homogeneity by measuring their width and crossover distance using JMicrovision software[2]. In addition to the previously reconstructed population with crossover distances between 130 and 150 nm (F140 population), we define an additional subpopulation characterized by crossover distances between 110 and 130 nm (F120 population). These fibrils were processed in the same way as the previous fibril reconstruction[1]. Fibrils were segmented using EMAN's BOXER[3]. Image processing was performed using the SPIDER software package[4] as described[1, 5]. As a first reference model, we chose a cylinder and obtained a second 3D structure of an Aβ(1-40) amyloid fibril from the F120 subset at a resolution of ~10 Å (image processing statistics are summarized in Supp. Table 1) (Supp. Figure 1A and 2). In addition, we also tested the F140 structure as a reference structure, which gave rise to a very similar cross-section (data not shown).

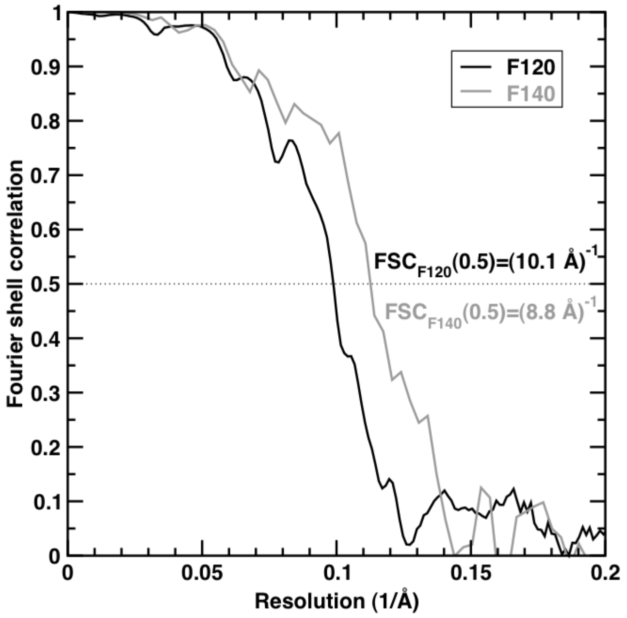


**Supp Figure 2.** Resolution assessment of F120 and F140 fibril populations. Fourier-shell correlation between two halves of the datasets are compared. F120 has been determined at a resolution of ~10 Å. Data for F140 fibrils were taken from Sachse et al.[1]

We compared the two subpopulations F120 and F140 by difference mapping. First, we aligned the two cross-sectional slices of the F120 and F140 reconstruction, and subsequently normalized their densities and finally subtracted F120 from F140 (Figure 3C).

**Supp. Table 1.** Image processing statistics of two Aβ(1-40) fibril populations. Data for F140 fibrils were taken from Sachse et al.[1]

| Fibril population | F120 | F140 |
|---|---|---|
| Resolution at FSC 0.5/0.143 (Å) | 10.1/8.7 | 8.8/7.1 |
| Total length of non-overlapping segments (nm) | 43,160 | 87,265 |
| Number of fibrils | 58 | 188 |
| Number of segments | 6129 | 11527 |
| Segment size (nm) | 84.2 | 84.2 |
| Size of 3D reconstruction (nm) | 77.7 | 77.7 |
| Segment step size (nm) | 6.3 | 6.3 |
| Average crossover distance/repeat distance (nm) | 121.0/0.48 | 142.5 / 0.48 |
| Pixel size on the specimen (Å) | 2.4 | 1.2 |

## *Determination of polar moment of inertia and moment of inertia*

### *a. Polar moment of inertia*

With the knowledge of the 3D fibril structure, we can estimate the resistance against torsional stress arising from the cross-sectional structure. Thus, we computed the polar moment of inertia $I_z$ of the cross-section excised from the 3D fibril cryo-EM reconstruction:

$$I_z = \int r^2 dA \qquad \text{(Supp. Eq. 1).}$$

where *r* is the radial distance and *A* is the cross-sectional area of a pixel. To evaluate the integral, the 3D reconstructions of F120 and F140 were filtered to a resolution of 20 Å to smooth the rendering of the cross-sectional density. Then, we converted the cross-sections into binary masks at the threshold of 3 x sigma. For each pixel with a density of one, we calculated the radial distance to the center of the cross-section, squared the distances and multiplied by the elemental pixel area of 1.2 x 1.2 Å. Finally, these products were added to yield the polar moment of inertia.

### *b. Moment of inertia*

The cross-sectional shape confers resistance against bending deformation, which is dependent on the axis where the force is applied. The moments of inertia $I_x$ and $I_y$ are defined as:

$$I_x = \int y^2 dA \qquad \text{(Supp. Eq. 2)}$$

and

$$I_y = \int x^2 dA \qquad \text{(Supp. Eq. 3)}$$

where *y* and *x* represent the distances of a pixel with area *dA* to the x- and y-axis, respectively (Figure 4B). Since Aβ(1-40) fibrils exhibit a regular twist, this directional dependence will be averaged over the course of a single crossover to:

$$I_{\overline{xy}} = \frac{I_x + I_y}{2} \qquad \text{(Supp. Eq. 4).}$$

For the polar moment of inertia, we calculated the distance *r* to the fibril axis, squared them and multiplied by the elemental pixel area of 1.2 x 1.2 Å. We used the same binary mask to calculate both moments. In agreement with Supp. Eq. 1 and Supp. Eq. 4, $I_{\overline{xy}}$ reduces to half of $I_z$.

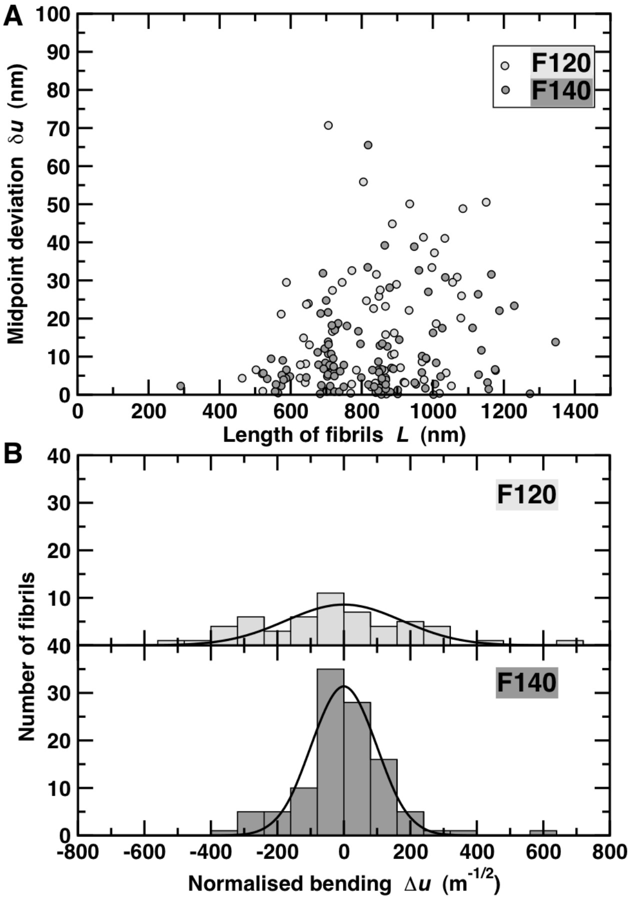


**Supp. Figure 3.** Flexibility analysis of 178 fibrils. Bending analysis. (A) Parameters of fibril length and corresponding midpoint deviation were measured for the two subpopulations F120 and F140 and plotted in a scatter diagram. (B) Two histograms of length-normalized bending were derived from the previous measurements of the two subpopulations and the entire population and subsequently fitted with Gaussian curves of a standard deviation of 174 and 96 $m^{1/2}$ respectively. While the different bending properties of F120 and F140 fibrils are statistically significant, this difference may be due to the flattened cross-sections of these fibrils. Since the bending of the fibrils is limited to the two-dimensional plane of the ice layer, it cannot be excluded that bending occurs predominantly at crossovers, i.e. where the flat side of a fibril is oriented perpendicular to the ice layer (Figure 1C). As a result, fibrils with a shorter crossover distances could possess a higher susceptibility to bending than fibrils with longer crossover distances.

## *Flexibility analysis*

A previous flexibility analysis on sickle-cell hemoglobin fibers based on raw cryo-EM images was proposed by Turner and co-workers[6]. The analysis assumes that fibrils consist of an isotropic and homogeneous material. In analogy, we determined twisting and bending parameters of Aβ(1-40) amyloid fibrils.

## *a. Torsion analysis*

***Supp. Table 2. Torsional properties of Aβ(1-40) fibrils***

| Fibril population | F120* | F140* |
|---|---|---|
| Crossover distance, *d,* range [nm] | 110 - 130 | 130 – 150 |
| Averaged mean crossover distance, $d$ ± averaged standard deviation $s_d$ [nm] | 122 ± 5.6 | 141 ± 7.0 |
| Torsional persistence length, $l_c$ $[10^{-6}$ m] | 9.3 (0.3) | 8.6 (0.1) |
| Torsional rigidity, $c$ $[10^{-26}$ $Nm^2]$ | 3.5 (0.1) | 3.3 (0.1) |
| Polar moment of inertia, $I_z$ $[10^{-33}$ $m^4]$ | 2.8 (0.9) | 2.6 (0.8) |
| Shear modulus, *G* [MPa] | 12.7 (2.6) | 12.7 (2.7) |

* error in parentheses

The distribution of crossover distances *d* provides information on the internal torsional properties of the fibrils. Therefore, crossover distance measurements were taken from 60 digitized micrographs using JMicrovision software[2] (Figure 1C). In order to minimize measurement errors, we included only those fibrils with more than six crossovers in the flexibility analysis. Finally, we took a total of 1180 measurements from 178 fibrils and calculated the mean crossover distance of each individual fibril and their corresponding standard deviation. The analysis revealed that the crossover distance variation within a single fibril is significantly smaller than the variation present in the entire fibril population (Figure 2A). As a result of the subdivision of the fibril population, the F120 dataset contained 58 fibrils, the F140 dataset consisted of 110 fibrils (Figure 2B) and 10 fibrils outside the analyzed crossover range were discarded. According to Turner et al., 2006[6], we obtain the torsional persistence length $l_c$ of each fibril:

$$l_c = \frac{d^3}{(s_d)^2 \cdot \pi^2} \qquad \text{(Supp. Eq. 5).}$$

Finally, we averaged the torsional persistence length of F120 and F140 fibril members. Their torsional rigidity *c* can be determined as:

$$c = l_c \cdot k_B \cdot T \qquad \text{(Supp. Eq. 6),}$$

where $k_B$ is the Boltzmann constant ($1.381\text{x}10^{-23}$ Nm/K), *T* the temperature of the sample. Therefore, $l_c$ and *c* describe the resistance of fibril structures towards torsional deformations.

For a uniform elastic medium, the shear modulus *G* represents a material-specific constant characterizing its intrinsic twisting ability is dependent on the ratio of torsional rigidity *c* and the polar moment of inertia $I_z$:

$$G = \frac{c}{I_z} \qquad \text{(Supp. Eq. 7).}$$

We display the figures used for the torsional analysis in the abovementioned equations in Supp. Table 2 calculated for fibril subpopulations F120 and F140. We obtained similar figures when fibrils are subdivided into four subpopulations.

## *b. Bending analysis*

***Supp. Table 3. Bending properties of Aβ(1-40) fibrils***

| Fibril population | F120* | F140* |
|---|---|---|
| Normalized bending fluctuation, $s_{\Delta u}$ [$m^{-1/2}$] | 174.3 (20.0) | 96.1 (19.8) |
| Persistence length, $l_p$ [$10^{-6}$ m] | 33 (1) | 108 (3) |
| Bending rigidity, $\kappa$ [$10^{-25}$ $Nm^2$] | 1.3 (0.06) | 4.1 (0.2) |
| Moment of inertia, $I_{\overline{xy}}$ [$10^{-33}$ $m^4$] | | |
| lower and upper bounds | 0.3 – 2.5 | 0.3 - 2.3 |
| average | 1.4 (0.5) | 1.3 (0.5) |
| Young's modulus, $Y$ [MPa] | | |
| lower and upper bounds | 50 – 443 | 178 – 1,620 |
| average | 90 (36) | 320 (124) |

* error in parentheses

The bending of fibrils correlates with its path deviation at fibril midpoint $\delta u$ from the distance or length $L$ that connects the two fibril ends (Figure 1C). In the analysis, we did not take into account out-of-the-plane fibril bending because of the confined fibril embedding in the ice layer and the small specimen tilt that we determined at ±12º using CTFTILT[7]. We measured the length $L$ and midpoint deviation $\delta u$ from the 178 fibrils; graphical analysis of these data pairs confirms that longer midpoint deviations are more commonly found in longer fibrils (Supp. Figure 3A). In analogy to Turner and co-workers[6, 8], we computed length-normalized bending fluctuations $\Delta u$ for each subpopulation F120 and F140 (Supp. Figure 3B) according to:

$$\Delta u = \frac{4\sqrt{3}}{L^{\frac{3}{2}}}\delta u \qquad \text{(Supp. Eq. 8).}$$

Since these thermal bending fluctuations are Gaussian distributed, the reciprocal of the mean squared deviation $(s_{\Delta u})^2$ corresponds to the persistence length $l_p$.

$$l_{p(3D)} = \frac{1}{(s_{\Delta u})^2} \qquad \text{(Supp. Eq. 9).}$$

Hence the bending rigidity $\kappa$ is given by:

$$\kappa = l_p \cdot k_B \cdot T \qquad \text{(Supp. Eq. 10).}$$

Therefore, $l_p$ and $\kappa$ reflect the resistance of fibrils towards bending. We calculated the material constant describing the resistance against bending or Young's modulus $Y$ by the ratio of the bending rigidity $\kappa$ and the cross-sectional moment of inertia $I_{\overline{xy}}$:

$$Y = \frac{\kappa}{I_{\overline{xy}}} \qquad \text{(Supp. Eq. 11).}$$

The figures used in the aforementioned equations characterizing the bending properties are summarized in Supp. Table 3. Because of the high anisotropy in cross section, we also estimated lower and upper bounds of the Young's modulus using $I_x$ and $I_y$ as the cross-sectional moment of inertia (Supp. Table 3). We estimated the errors in Supp. Table 2 and 3 according to the laws of error propagation and assumed a primary measurement error of 2 nm.

[1] C. Sachse, M. Fändrich & N. Grigorieff, *Proc. Natl. Acad. Sci. U.S.A.* **2008**, Paired beta-sheet structure of an Abeta(1-40) amyloid fibril revealed by electron microscopy, 105, 7462-7466.

[2] N. Roduit, *Geophysical Research Abstracts* **2006**, Quantification and measurement in digital images of thin sections with JMicroVision,

[3] S. J. Ludtke, P. R. Baldwin & W. Chiu, *J Struct Biol* **1999**, EMAN: semiautomated software for high-resolution single-particle reconstructions, 128, 82-97.

[4] J. Frank, M. Radermacher, P. Penczek, J. Zhu, Y. Li, M. Ladjadj & A. Leith, *J Struct Biol* **1996**, SPIDER and WEB: processing and visualization of images in 3D electron microscopy and related fields, 116, 190-199.

[5] C. Sachse, J. Z. Chen, P. D. Coureux, M. E. Stroupe, M. Fändrich & N. Grigorieff, *J. Mol. Biol.* **2007**, High-resolution electron microscopy of helical specimens: a fresh look at tobacco mosaic virus, 371, 812-835.

[6] M. S. Turner, R. W. Briehl, J. C. Wang, F. A. Ferrone & R. Josephs, *J. Mol. Biol.* **2006**, Anisotropy in sickle hemoglobin fibers from variations in bending and twist, 357, 1422-1427.

[7] J. A. Mindell & N. Grigorieff, *J Struct Biol* **2003**, Accurate determination of local defocus and specimen tilt in electron microscopy, 142, 334-347.

[8] J. C. Wang, M. S. Turner, G. Agarwal, S. Kwong, R. Josephs, F. A. Ferrone & R. W. Briehl, *J. Mol. Biol.* **2002**, Micromechanics of isolated sickle cell hemoglobin fibers: bending moduli and persistence lengths, 315, 601-612.

[9] J. F. Smith, T. P. Knowles, C. M. Dobson, C. E. Macphee & M. E. Welland, *Proc. Natl. Acad. Sci. U.S.A.* **2006**, Characterization of the nanoscale properties of individual amyloid fibrils, 103, 15806-15811.
